# High prevalence of thrombophilic risk factors in patients with central retinal artery occlusion

**DOI:** 10.1186/s12959-023-00525-z

**Published:** 2023-07-28

**Authors:** Radosław Dziedzic, Lech Zaręba, Teresa Iwaniec, Agnieszka Kubicka-Trząska, Bożena Romanowska-Dixon, Stanisława Bazan-Socha, Jerzy Dropiński

**Affiliations:** 1 Doctoral School of Medical and Health Sciences, Jagiellonian University Medical College, Łazarza 16, Krakow, 31-530 Poland; 2 Department of Internal Medicine, Faculty of Medicine, Jagiellonian University Medical College, Jakubowskiego 2, Krakow, 30-688 Poland; 3 College of Natural Sciences, Institute of Computer Science, University of Rzeszow, Pigonia 1, Rzeszow, 35-310 Poland; 4 Department of Hematology, Jagiellonian University Medical College, Kopernika 17, Krakow, 31-501 Poland; 5 Faculty of Medicine, Department of Ophthalmology, Clinic of Ophthalmology and Ocular Oncology of University Hospital, Jagiellonian University Medical College, Kopernika 38, Krakow, 31-501 Poland

**Keywords:** Central retinal artery occlusion, CRAO, Thrombophilia, Thrombosis, Risk factors, Intima-media thickness

## Abstract

**Introduction:**

Central retinal artery occlusion (CRAO) is a common cause of blindness and visual morbidity. In the majority of cases, it is related to thrombotic embolism. Nevertheless, the role of inherited or acquired thrombophilic risk factors in CRAO pathogenesis has not been comprehensively studied.

**Methods:**

In 126 CRAO patients (66 [52.4%] men, median age 55 [range: 18–80] years) and 107 matched controls (56 [52.3%] men, median age 53 [range: 34–78] years) we evaluated classical atherosclerotic risk factors, including serum lipid profile and glucose level, analyzed intima-media complex thickness (IMT) of external carotid arteries, and performed transthoracic echocardiography. Furthermore, we established the prevalence of inherited and acquired thrombophilic risk factors, such as factor V Leiden (FVL) and prothrombin 20210 G/A genetic variants, plasma activity of factor (F) VIII, protein C and antithrombin activity, and free protein S levels. We also assessed the presence of antiphospholipid antibodies (APLA) and evaluated blood homocysteine in all enrolled subjects. Additionally, we estimated the occurrence of Val34Leu polymorphism of the A subunit of coagulation factor XIII (FXIII-A) in both groups as a potential thrombosis-protecting factor.

**Results:**

Among traditional atherosclerotic risk components, obesity/overweight and hypercholesterolemia were the most common in the CRAO group and occurred in 103 (81.7%) and 85 (67.5%) patients, respectively. CRAO patients also had elevated IMT and altered echocardiographic parameters, indicating diastolic cardiac dysfunction. In thrombophilia investigations, at least one laboratory risk factor occurred in 72.2% (n = 91) of CRAO patients, with APLA as the most frequent, detected in 38.1% (n = 48) of them (almost seven times more frequent than in controls, p < 0.001). Deficiencies in protein C activity and free protein S levels were also common in the CRAO group, reported in 17.5% (n = 22) and 19.8% (n = 25) of patients, respectively. Interestingly, among two analyzed prothrombotic genetic variants, only the FVL was related to CRAO, with the allelic frequency 2.4 times more prevalent than in controls (p = 0.044). Finally, the CRAO group was characterized by hyperhomocysteinemia, almost twice as common as in controls (p = 0.026). Antithrombin deficiency, elevated FVIII, and FXIII-A Val34Leu polymorphism were not associated with CRAO.

**Conclusions:**

Our findings suggest that thrombophilia plays a vital role in the pathogenesis of CRAO. Thus, proper laboratory screening should be considered in the primary and secondary prevention of those episodes, with implementing appropriate therapy as needed.

## Introduction

Central retinal artery occlusion (CRAO) is an ophthalmic condition in which the retinal circulation is impaired, mainly due to the presence of a thrombus in the central retinal artery, the most common underlying cause of its obstruction, leading to a sudden visual loss [1]. The pathogenesis of CRAO is still not fully understood; however, traditional cardiovascular risk factors, including hypertension, diabetes mellitus, and dyslipidemia, are believed to play an essential role in its development [2]. Similarly, other hereditary or acquired laboratory thrombophilic risk factors contribute to the pathogenesis of CRAO [3]. Furthermore, CRAO patients are more likely to develop future cardiovascular events, including stroke and myocardial infarction, and have a higher risk of all-cause mortality [4, 5]. Therefore, CRAO should be considered as a severe threat to patients, and identifying factors referring most to that danger is of critical importance. Moreover, prompt management in the first hours after a CRAO episode, including intravenous alteplase treatment, is crucial for maintaining vision [6].

Among well-recognized thrombophilic risk factors, one of the most important are inherited ones [7, 8], including factor V Leiden (FVL) and prothrombin G20210A genetic variants and a deficiency in the activity of natural anticoagulant proteins. FVL precludes active factor V inactivation by the protein C and is associated with venous thromboembolism (VTE), either in first or recurrent episodes [9]. In turn, prothrombin G20210A is a genetic condition that increases the stability of factor II transcripts, favoring its enhanced translation with increased plasma concentration [10]. Deficiencies of protein C and S activity lead to disturbances in the inactivation of active factors V and VIII, enhancing the coagulation cascade [11]. Antithrombin deficiency is associated with a lower ability to inhibit thrombosis on several steps of the coagulation cascade, resulting in much higher prothrombotic risk [12]. Another important hypercoagulable and cardiovascular risk factor is hyperhomocysteinemia, which decreases adenosine production by endothelium and enhances local thromboxane generation with platelet aggregation [13].

In turn, among acquired thrombophilic risk factors, the most important is the presence of antiphospholipid antibodies (APLA), a heterogeneous family of autoantibodies directed against phospholipids and phospholipid binding proteins. They are evaluated according to the Sapporo criteria by coagulometric assay (lupus anticoagulant [LA]) and by ELISA tests (anticardiolipin [ACL] and anti-beta-2-glycoprotein I [Aβ2GPI] antibodies, classes IgM and IgG) [14]. APLA may be associated with venous and arterial thrombosis, recurrent miscarriages, and thrombocytopenia, which clinically are recorded as antiphospholipid syndrome (APS) [15, 16].

Previously, the involvement of hereditary and acquired thrombophilia has been demonstrated in the pathogenesis of arterial and venous retinal vascular occlusions [17, 18]. Furthermore, there are several reports, specifically in CRAO, describing the higher prevalence of different thrombophilic risk factors in those patients [3, 19–25]. However, those studies usually comprised a small group of patients and only little explored associations between thrombophilia and clinical characteristics. Furthermore, their results are ambiguous; thus, it is uncertain whether thrombophilia screening should be recommended in secondary prevention after CRAO development. On the other hand, patients with thrombophilia are prone to develop other thromboembolic complications after a first CRAO event [26]. Therefore, thrombophilia-related disorders and possibly thrombophilic factors need to be investigated after CRAO episodes.

The aim of this study was to analyze the thrombophilic risk factors in a group of patients with CRAO. We also evaluated the relationship between these factors and carotid intima-media thickness (IMT), referring to the progression of atherosclerosis [27], presence of atherosclerotic plaques in carotid arteries, and transthoracic echocardiography (TTE) parameters, which are beneficial in the general evaluation of the cardiovascular system [28]. In addition, we also analyzed the prevalence of other traditional cardiovascular risk factors in the CRAO group, including arterial hypertension, diabetes, obesity, dyslipidemia, and smoking. To our knowledge, this is the first study to determine the most essential thrombophilic risk factors in a large group of patients with CRAO.

## Patients and methods

### Study design and population

The following research provides single-center data and has a case-control type, and was approved by the Bioethics Committee of the Jagiellonian University Medical College (permit No: KBET/79/B/2013). The study procedures were carried out under the ethical guidelines of the Declaration of Helsinki. All participants gave informed consent in writing to participate in the study.

The study group comprised 126 consecutive adult participants (male/female: 66/60, median age: 55, range 18 to 80 years) with CRAO episodes who visited the Outpatient Clinic of the Department of Allergy and Clinical Immunology, University Hospital, Krakow, Poland, from 2013 to 2019 for thrombophilia screening. Patients were previously diagnosed and treated in the Department of Ophthalmology, Clinic of Ophthalmology and Ocular Oncology, Jagiellonian University Medical College, Krakow, Poland. An experienced ophthalmologist made the diagnosis of CRAO, based on a patient’s history of a sudden painless visual loss in one eye and fundoscopic criteria, including retinal whitening with a “cherry-red spot” appearance of macula and segmental blood flow in retinal blood vessels [29]. The time interval between the onset of ocular symptoms and the ophthalmological examination ranged from 2 h to 5 days in the analyzed group of patients. Baseline ophthalmic examination revealed in the affected eye, the best-corrected visual acuity (BCVA) ranged from light perception to hand motions, and the mean intraocular pressure: was 17.4 mmHg (ranging from 12 to 22 mmHg). In all cases, the fundoscopy showed a pale fundus with a “cherry-red spot” in the macula with the attenuation of the retinal arterioles and the segmentation of blood flow. The ophthalmic treatment in patients with occlusion of fewer than 48 h duration at presentation included procedures to lower the intraocular pressure with topical therapy (beta-blockers, carbonic anhydrase inhibitors), intravenous acetazolamide, ocular massage and/or anterior chamber paracentesis to move the embolus away from the central retina. To increase retinal blood flow by vasodilatation the increased carbon dioxide concentration mixture for breathing was also used. All patients were referred to the internal medicine specialist for further immediate work-out and systemic management. Fibrinolytic therapy was introduced in 7 (5.6%) cases of CRAO. The thrombophilia screening and basic laboratory tests were performed at least three months after the CRAO event. At enrollment, all patients with CRAO history received 75–150 mg daily aspirin.

The control group consisted of 107 subjects (male/female: 56/51, median age: 53, range 34–78 years) matched by sex, age, and body mass index (BMI). They had no history of eye diseases other than refractive errors and were recruited from hospital personnel. Due to planned genetic testing, we enrolled only unrelated persons in the control group.

Exclusion criteria for both groups included: acute illness in the last six months, a history of myocardial infarction, stroke, atrial fibrillation, or venous thromboembolism, current anticoagulant therapy, ongoing cancer treatment, known active autoimmune or hematologic disease, carotid artery dissection or presence of blood clots in heart chambers, liver injury (elevated serum alanine aminotransferase levels more than twice the upper limit of the reference range), and kidney failure (estimated glomerular filtration rate less than 60 ml/min/1.73 m^2^). Patients with hypertension, diabetes mellitus, and hypercholesterolemia were eligible. Arterial hypertension was defined as a history of blood pressure greater than 140/90 mm Hg or present antihypertensive treatment. Hypercholesterolemia was defined as serum total cholesterol greater than 5.2 mmol/l or current statin treatment. Diabetes mellitus was defined as fasting serum glucose above 7.0 mmol/l or the current use of insulin/hypoglycemic agents. Obesity/overweight was defined as values of body mass index (BMI) of at least 30.0 kg/m^2^ and 25.0 kg/m^2^ to less than 30.0 kg/m^2^, respectively. Smoking habit was defined as the use of at least one cigarette per day. The family history of cardiovascular diseases (CVD) was positive in patients with confirmed CVD in a first-degree relative, including arterial hypertension, coronary artery disease, atherosclerosis, heart attack, rhythm disorders, heart failure, venous thrombosis, or stroke.

Overall, we have included all consecutive adult patients with CRAO established in an eye examination who did not meet the mentioned above exclusion criteria.

### Laboratory analysis

Fasting blood samples were drawn from the ulnar vein between 7 a.m. and 9 a.m. with minimal tourniquet use after sufficient rest. The blood samples were kept in tubes with 0.109 mol/l sodium citrate (vol/vol, 9:1) centrifuged at 2000 x g for 10 min at room temperature within two hours of collection; plasma was frozen in aliquots and stored at − 70 °C until tested.

### Basic laboratory test

Complete blood cell count, creatinine, glucose, and lipid profile were assessed using routine laboratory techniques. The C-reactive protein (CRP) level was evaluated using the Johnson & Johnson VITROS 250.

### Ultrasound studies

The methods of ultrasound examinations in CRAO patients were described in detail in our previous paper [30]. Here, intima-media thickness (IMT) of the common carotid artery, the presence of atherosclerotic plaques, and also a transthoracic echocardiogram (TTE), were used for analysis to look for correlations between them and thrombophilic risk factors. Briefly, IMTs of the common carotid artery were evaluated on both sides of the longitudinal projection immediately proximal to its bifurcation using a 10 MHz linear transducer. Furthermore, results from TTE, including left ventricular ejection fraction (LVEF), systolic pulmonary artery pressure (SPAP), and other basic echocardiographic parameters, were recorded.

### Coagulation studies

The activity of protein C and antithrombin were measured using chromogenic methods (Berichrom Protein C, Berichrom Antithrombin; Siemens, Marburg, Germany). Levels of free protein S were evaluated with turbidimetric assay (Innovance Free protein S; Siemens, Marburg, Germany). Factor VIII activity was analyzed by a coagulometric test (Siemens, Marburg, Germany). The homocysteine concentration was assessed with a chemiluminescent microparticle immunoassay.

### Measurements of antiphospholipid antibodies

Lupus anticoagulant (LA) was evaluated using a three-step procedure according to the guidelines of The International Society of Thrombosis and Haemostasis [31]. To clarify, dilute Russell’s viper venom time (dRVVT; LA1-screen; Siemens, Germany) and a sensitive aPTT (PTT LA; Diagnostica Stago, France) were used for screening, whereas LA2-confirm (Siemens, Germany) and Staclot LA (Diagnostica Stago, USA) were run to affirm the result. Levels of anticardiolipin (ACL) and anti-β2-glycoprotein I (Aβ2GPI) antibodies, IgM and IgG classes, were performed with commercially available immunoenzymatic assays (QUANTA Lite® aCL and aβ2GPI (Inova Diagnostics, San Diego, USA)).

### Genetic analysis

Genotyping for factor V Leiden R506Q (mutation rs6025) (FVL) and prothrombin G20210A (mutation rs1799963) was performed with TaqMan assays (Applied Biosystems), as reported elsewhere [32, 33]. The factor(F)XIII-A Val34Leu polymorphism was investigated with real-time polymerase chain reaction [34].

### Statistical elaboration

Statistical analyses were performed using STATISTICA Tibco v. 13.3 software (StatSoft, Tulsa, USA). The Shapiro-Wilk test was used to evaluate data distribution. Continuous variables in groups were non-normally distributed, thus, were shown as median with interquartile range (IQR). Categorical variables were given as numbers with percentages. The Chi^2^ test or exact Fisher test were used to compare categorical data. Univariate linear regression models (beta coefficient and 95% confidence interval) with adjustment for age, sex and BMI were performed to evaluate the relationship between the two selected parameters. The occurrence of thrombophilic risk factors with a cut-off point of 50 years was analyzed in some cases. Independent determinants of the relative increase in flow-mediated dilatation of the brachial artery were established in multivariate linear regression models, built using a stepwise forward selection procedure, verified by Snedecor’s F-distribution. The R^2^ was checked as a measure of variance. A p-value less than 0.05 was considered statistically significant.

## Results

### Patients and controls

Demographic and clinical characteristics of the CRAO group and controls have been provided in Table 1. As it has been shown, both groups were similar according to age, sex, and BMI. Nevertheless, patients with CRAO had more frequent arterial hypertension, diabetes mellitus, and hypercholesterolemia. Furthermore, they were characterized by a higher prevalence of obesity/overweight and smoked more often than controls. Additionally, in the CRAO group, we documented more frequent positive family history of CVD than in controls.

**Table 1:** Demographic and clinical characteristics and basic laboratory tests in patients with central retinal artery occlusion and controls

Parameter	Patients n = 126	Controls n = 107	p-value
Demographic characteristics			
Age, years	55.7 (53.7–57.7)	53.7 (51.9–55.5)	0.1
Sex, male, n (%)	66 (52.4%)	56 (52.3%)	0.9
Body mass index, kg/m^2^	26.6 (25.5–28.3)	26.4 (24.2–27.9)	0.1
Clinical characteristics			
Hypertension, n (%)	49 (38.9%)	27 (25.2%)	0.038*
Diabetes mellitus, n (%)	24 (19.0%)	9 (8.4%)	0.012*
Hypercholesterolemia, n (%)	85 (67.5%)	46 (43.0%)	< 0.001*
Obesity/overweight, n (%)	103 (81.7%)	65 (60.7%)	< 0.001*
Smoking habit, n (%)	47 (37.3%)	23 (21.5%)	0.015*
Positive family history of CVD, n (%)	46 (36.5%)	20 (18.7%)	0.004*
Laboratory tests			
Hemoglobin, g/dl	14.5 (13.6–15.4)	13.9 (12.8–14.8)	< 0.001*
White blood cells, 10^3^/µL	6.8 (5.3–7.9)	6.1 (5.0–7.1)	0.011*
Blood platelets, 10^3^/µL	228 (190–276)	228 (198–278)	0.86
Glucose, mmol/l	5.7 (4.9–6.1)	5.4 (4.9–5.8)	0.10
Total cholesterol, mmol/l	5.4 (4.9–5.9)	5.0 (4.6–5.5)	< 0.001*
High-density lipoprotein cholesterol, mmol/l	1.2 (1.1–1.4)	1.4 (1.2–1.6)	< 0.001*
Low-density lipoprotein cholesterol, mmol/l	3.2 (2.9–3.7)	3.1 (2.8–3.4)	0.13
Triglycerides, mmol/l	1.8 (1.4–2.1)	1.7 (0.9–1.9)	< 0.001*
Creatinine, µmol/l	88.3 (78.0–98.0)	79.2 (69.3–92.1)	< 0.001*
C-reactive protein, mg/l	4.8 (3.6–6.5)	2.8 (1.8–3.9)	< 0.001*

Categorical variables are presented as numbers (percentages) and continuous variables as median with interquartile range. The statistically significant results are marked with an asterisk.

### Basic laboratory tests

Table 1 also presents the results of basic laboratory tests. As it has been shown, higher white blood cell count and hemoglobin levels characterized the CRAO group. According to the lipid profile, patients had elevated total cholesterol and triglyceride levels and lower high-density lipoprotein cholesterol. Furthermore, they had slightly higher serum creatinine and CRP concentrations.

### Prevalence of thrombophilic risk factors in the CRAO group and controls

The prevalence of thrombophilic risk factors, as the percentage of subjects above/below the reference ranges in the patient and control groups, are provided in Table 2. On the other hand, Table 3 demonstrates results as specific values measured in laboratory investigations.

The most often detected thrombophilia-related risk factor was the occurrence of APLA, including LA, ACL and/or Aβ2GPI antibodies, stated in more than one-third of the CRAO cohort. Interestingly, the positive result for any APLA test was detected 6.8 times more frequently in the CRAO group than in controls (n = 48 [38.1%] vs. 6 [5.6%], p < 0.001). Moreover, positive results for ACL antibodies were reported much more in CRAO patients (n = 20 [15.9%] vs. n = 1 [0.9%], p < 0.001); likewise, the presence of Aβ2GPI antibodies was overwhelmingly more common in patients (n = 31 [24.6%] vs. n = 2 [1.6%], p < 0.001). LA was documented in 6 CRAO patients (1 man and 5 women) and 3 individuals from a control group (p = 0.51). According to the APLA presence (+ APLA) in the CRAO group, there was no difference regarding sex (+ APLA in 41 men [62.1%] vs. +APLA in 37 women [61.7%], p = 0.96) and age with 50 years as a cut-off point (+ APLA in 58 patients with ≥ 50 years old [65.9%] vs. +APLA in 20 patients < 50 years old [52.6%], p = 0.16). No patients had triple-positive APLA (LA and ACL and Aβ2GPI). However, we noticed 9 CRAO patients with at least two different types of APLA (LA and/or ACL and/or Aβ2GPI), with none in the control group (p = 0.004).

Furthermore, the CRAO group was characterized by a higher rate of protein C activity and free protein S level deficiency (n = 22 [17.5%] vs. n = 2 [1.9%], p < 0.001; n = 25 [19.8%] vs. n = 7 [6.5%], p = 0.003; respectively). Hyperhomocysteinemia was detected almost 2-times more frequently in CRAO patients than in the control group (n = 28 [22.2%] vs. n = 12 [11.2%], p = 0.026).

The occurrence of antithrombin deficiency and higher FVIII were comparable between both groups.

Interestingly, FVL but not PT 20,210, significantly more often occurred in the CRAO group than in control individuals. FVL was documented in 17 CRAO patients, with heterozygosity in 14 individuals and homozygosity in 3 subjects. There were no individuals with both mutations (FVL and FII G20210A).

Altogether, at least one out of the twelve thrombophilic risk factors evaluated in laboratory investigations was observed in 91 (72.2%) CRAO patients. Only one was detected in 34 (27.0%) patients, two in 21 (16.7%), and 18 (14.3%) had three. Interestingly, 14.3% (n = 18) of patients simultaneously had more than three thrombophilic risk factors.

Sex, age (50 years as a cut-off point), and family history of cardiovascular diseases did not impact our result.

**Table 2:** Prevalence of thrombophilic risk factors in patients with central retinal artery occlusion and controls. Data presented as numbers and percentages of patients with results above/below the reference range

Thrombophilic risk factor	CRAO group n = 126	Control group n = 107	p-value
Protein C activity (< 75%), n (%)	22 (17.5%)	2 (1.9%)	< 0.001*
Free protein S level (< 75%), n (%)	25 (19.8%)	7 (6.5%)	0.003*
Antithrombin activity (< 75%), n (%)	10 (7.9%)	4 (3.7%)	0.18
Factor VIII activity (> 150%), n (%)	26 (20.6%)	18 (16.8%)	0.46
Homocysteine (> 15 µmol/l), n (%)	28 (22.2%)	12 (11.2%)	0.026*
Anticardiolipin antibodies IgM (> 20 MPL), n (%)	13 (10.3%)	1 (0.9%)	0.003*
Anticardiolipin antibodies IgG (> 15 GPL), n (%)	17 (13.5%)	0 (0%)	< 0.001*
Anti-beta-2-glycoprotein I antibodies IgM (> 20 SMU), n (%)	16 (12.7%)	1 (0.9%)	< 0.001*
Anti-beta-2-glycoprotein I antibodies IgG (> 15 SGU), n (%)	25 (19.8%)	1 (0.9%)	< 0.001*
Lupus anticoagulant, n (%)	6 (4.8%)	3 (2.8%)	0.51
Factor V Leiden mutation, n (%)	heterozygosity 14 (11.1%) homozygosity 3 (2.4%) altogether 17 (13.5%)	heterozygosity 4 (3.7%) homozygosity 2 (1.9%) altogether 6 (5.6%)	0.044*
Factor II G20210A mutation, n (%)	heterozygosity 12 (9.5%) homozygosity 1 (0.8%) altogether 13 (10.3%)	heterozygosity 3 (2.8%) homozygosity 2 (1.9%) altogether 5 (4.7%)	0.11

Categorical variables are presented as numbers (percentages). The statistically significant results are marked with an asterisk.

**Table 3:** Levels of thrombophilic risk factors in patients with central retinal artery occlusion and controls

Thrombophilic risk factor	CRAO group n = 126	Control group n = 107	p-value
Protein C activity, %	102.2 (82.1–117.7)	104.8 (89.5–124.8)	0.17
Free protein S level, %	90.7 (78.2–111.8)	90.2 (80.3–106.8)	0.66
Antithrombin activity, %	98.9 (86.4–111.1)	96.8 (82.4–108.6)	0.62
Factor VIII activity, %	121.6 (103.3–147.7)	108.9 (90.6–138.4)	0.016*
Homocysteine, µmol/l	13.1 (10.2–15.2)	10.6 (9.4–13.5)	< 0.001*
Anticardiolipin antibodies IgM, MPL	8.2 (4.1–14.9)	6.4 (4.5–7.9)	< 0.001*
Anticardiolipin antibodies IgG, GPL	7.9 (3.5–12.5)	5.4 (3.5–7.7)	< 0.001*
Anti-beta-2-glycoprotein I antibodies IgM, SMU	11.4 (7.2–16.2)	6.8 (4.9–8.9)	< 0.001*
Anti-beta-2-glycoprotein I antibodies IgG, SGU	10.9 (7.4–13.9)	6.9 (4.9–8.6)	< 0.001*

Continuous variables are presented as median with interquartile range. The statistically significant results are marked with an asterisk

### Factor XIII Val34Leu polymorphism in the CRAO group and controls

The frequency of the Val/Val, Val/Leu, and Leu/Leu variants in the CRAO group (n = 63) was 47.6%, 41.3%, and 11.1%, respectively. The corresponding numbers in the control group (n = 68) were 48.5%, 38.2%, and 13.2%, respectively. The distribution of genotypes did not show a significant difference between the two groups (p = 0.90).

### Ultrasound investigation in CRAO patients and controls

A summary of ultrasound investigations is presented in Table 4. As it has been shown, the CRAO group was characterized by a 23.1% increased mean value of IMT of the common carotid artery. Furthermore, the study group had a slightly decreased left ventricular ejection fraction and a 10.0% increased thickness of the interventricular septum and left ventricle posterior wall. Also, the left ventricle end-diastolic and end-systolic dimensions were higher in the CRAO group (increase in 3.6% and 3.3%, respectively), with elevated pulmonary artery systolic pressure.

Additionally, in CRAO patients, we observed a high prevalence of degenerative heart valve lesions (n = 81, [64.3%]) and atherosclerotic plaques in the common carotid arteries or the initial external/internal carotid arteries sections (n = 42, [33.3%]). Interestingly, CRAO patients with atherosclerotic plaques more frequently had at least three laboratory-evaluated thrombophilic risk factors than the remaining (n = 20, [47.6%] vs. n = 16, [19.0%], p < 0.001). The presence of degenerative heart valve lesions was unrelated to the analyzed thrombophilic risk factors.

**Table 4:** Intima-media thickness and basic echocardiographic parameters in patients with central retinal artery occlusion and controls

Parameter	Patients n = 126	Controls n = 107	p-value
Ultrasound parameter of atherosclerosis			
Mean value of the intima-media thickness of a common carotid artery, mm	0.80 (0.69–0.98)	0.65 (0.58–0.73)	< 0.001*
Echocardiographic parameters			
Left ventricular ejection fraction, %	67 (65–69)	68 (66–70)	0.026*
Interventricular septum thickness, cm	1.1 (1.0–1.4)	1.0 (0.9–1.1)	< 0.001*
Left ventricle posterior wall thickness, cm	1.1 (0.9–1.2)	1.0 (0.9–1.0)	< 0.001*
Tricuspid regurgitation velocity, m/s	2.6 (2.4–2.9)	2.4 (2.2–2.7)	< 0.001*
Pulmonary artery systolic pressure, mm Hg	33.1 (31.9–34.3)	28.7 (27.7–29.6)	< 0.001*
Left ventricle end-diastolic dimension, mm	49.5 (48.7–50.2)	47.8 (47.2–48.5)	0.008*
Left ventricle end-systolic dimension, mm	31.0 (29.0–33.0)	30.0 (29.0–32.0)	0.011*

Categorical variables are presented as numbers (percentages), continuous variables as median and interquartile range. The statistically significant results are marked with an asterisk

### Relation of ultrasound parameters to thrombophilia

Next, we evaluated correlations of IMT and basic echocardiographic parameters with analyzed thrombophilic parameters. Interestingly, IMT in univariate linear regression models was related to the ACL antibodies in both classes (for IgM class: β = 0.31, 95% CI: 0.22 to 0.40; for IgG class: β = 0.34, 95% CI: 0.25 to 0.43), but also to the activity of factor VIII (β = 0.45, 95% CI: 0.37 to 0.54). Moreover, in a multiple regression model, among thrombophilic risk factors, elevated FVIII, anticardiolipin antibodies in the IgG class, free protein S level, protein C activity, and homocysteine predicted greater IMT values (Table 5). However, all the mentioned variables explained only 27% of the IMT variability.

**Table 5:** Multiple linear regression model for a relative increase of intima-media thickness (IMT) of the common carotid artery in the central retinal artery occlusion patients. Presented variables have been reported as independent determinants, explaining 27% of IMT variability

	β (95% CI)	R^2^
Intima-media thickness of the common carotid artery, mm		
Factor VIII, %	0.40 (0.30 to 0.49)	0.27
Anticardiolipin antibodies IgG, GPL	0.35 (0.24 to 0.45)	
Free protein S level, %	0.20 (0.09 to 0.31)	
Protein C activity, %	0.18 (0.06 to 0.29)	
Homocysteine, µmol/l	0.12 (0.02 to 0.21)	
Adjustment statistics	F = 11.70, p < 0.0001	

The resulting standardized regression coefficient (β) with a 95% confidence interval (95% CI) for a factor (independent variable) indicates the increase or decrease in standard deviations (SDs) of a dependent variable (IMT), when that particular factor increases with 1 SD and all other variables in the model remain unchanged

Interestingly TTE parameters describing thicker heart walls correlated positively with several thrombophilic risk factors. For example, the interventricular septum was associated with free protein S levels (β = 0.22, 95% CI: 0.13 to 0.31), ACL antibodies in IgM class (β = 0.19, 95% CI: 0.10 to 0.28), and factor VIII activity (β = 0.22, 95% CI: 0.13 to 0.30), after adjustment for potential confounders. In addition, factor VIII correlated with left ventricle posterior wall thickness (β = 0.18, 95% CI: 0.10 to 0.27).

Moreover, according to the pulmonary artery systolic pressure, we noticed relationships with protein C activity (β = − 0.23, 95% CI: − 0.33 to − 0.14), but also ACL (for IgG: β = 0.30, 95% CI: 0.21 to 0.40; for IgM: β = 0.20, 95% CI: 0.10 to 0.29) and Aβ2GPI antibodies (for IgG: β = 0.22, 95% CI: 0.13 to 0.31; for IgM: β = 0.18, 95% CI: 0.09 to 0.27) in both classes.

## Discussion

The present study demonstrates that CRAO is related to the laboratory-investigated thrombophilic risk factors; at least one was reported in 72.2% of CRAO patients, while at least three in almost 30%. Most frequently, we documented lower protein C activity and decreased free protein S levels, accompanied by positive results for APLA. Moreover, the allelic frequency of FVL was higher in the patient group, likewise to the presence of hyperhomocysteinemia. On the contrary, antithrombin deficiency, elevated FVIII, and FXIII-A Val34Leu polymorphism were not associated with CRAO.

To date, there are several reports on the role of thrombophilia in the pathogenesis of CRAO [3, 19–25]. However, these studies focused mainly on a small group of patients and usually considered branch and central retinal artery occlusion together. To our knowledge, this is the first original study to evaluate all the essential thrombophilic risk factors in such a large group of 126 patients with CRAO.

In addition, we analyzed the traditional cardiovascular risk factors reported in the majority of CRAO patients either, with the most prevalent of obesity/overweight, hypercholesterolemia, hypertension, and cigarette smoking, which confirmes their important role in CRAO pathogenesis. Therefore, it is essential in primary and secondary prevention of cardiovascular events to modify these risk factors with a healthy lifestyle and alternatively by medications. The results of ultrasound studies showed that increased intima-media thickness with some parameters of diastolic dysfunction of the left ventricle was observed in the CRAO patients more frequently. Thus, easy diagnostic methods such as echocardiography and carotid ultrasonography with intima-media thickness measurement may be helpful in early prophylactic procedures to prevent CRAO [30]. Moreover, it is worth highlighting that several thrombophilic risk factors showed a relationship with intima-media thickness, a prognostic marker of atherosclerosis, but also were associated with the presence of atherosclerotic plaques in carotid arteries and TTE parameters, routinely used in cardiovascular system evaluation.

Among studies analyzing thrombophilic risk factors in ophthalmologic vascular complications, particular attention deserves a meta-analysis by Romiti et al. [35]. It summarizes the prevalence of the most important inherited and acquired risk factors for thrombophilia retinal vascular occlusion (RVO) patients, documenting similar prevalences of inherited and acquired thrombophilias in RVO patients. However, in this analysis, only eleven studies on retinal artery occlusion (RAO) were included, which usually comprised a small group of patients with branch and central retinal artery occlusion together, limiting the interpretation regarding CRAO.

In our data, CRAO patients also had a higher incidence of decreased protein C activity and lowered free protein S levels than the control group. On the contrary, antithrombin activity was unchanged. Thus, our results contrast with published work on RAO patients, where no differences in the natural anticoagulants were noted [19] or even no detection of these factors in the patient group was documented [3, 25]. Congruent with the present study, the overall prevalence of natural anticoagulants deficiency was much higher than pooled estimated in a recent meta-analysis on RAO patients, such as 1%, 2% and 3% for deficiency of protein S, protein C and antithrombin, respectively [35]. However, our study represents CRAO patients only and the results might not be consistent as the reported for both branch and central RAO groups. Additionally, some differences in the studies also concerned the methodology of measuring the concentration or activity of proteins C and S, e.g., the total or free protein S levels.

According to the APLA, in our study, at least one (ACL, Aβ2GPI, or LA) was positive in almost 40% of CRAO patients. Several studies confirmed the higher prevalence of APL antibodies in RAO [3, 21], but evidence on CRAO is scarce. Interestingly, a survey by Palmowski-Wolfe et al. [21] evaluated the prevalence of APLA in patients with RVO, in which the occurrence of these antibodies was noted approximately one in five CRAO patients. However, there are also studies in which the prevalence of APLA, specifically ACL antibodies, is similar, considering CRAO patients and controls [19, 25]. On the other hand, the occurrence of lupus anticoagulant in our study group was similar to controls. Surprisingly, it contrasts with other studies, where the prevalence of LA was significantly higher in the RAO patients [3], positive in even more than 25% of them [19]. This discrepancy might be associated with the methodology of LA evaluation since the older studies might overestimate positive results. Intriguingly, the prevalence of APLA in RAO patients in a recent meta-analysis by Romiti et al. [35] calculated with a fixed effect model is around 17%, thus lower than in our study. Interestingly, in thrombotic pathogenesis, APLA play a complex role. They react with anionic or polar phospholipids such as cardiolipin and phospholipid-binding proteins [36]. Furthermore, APLA might inhibit the anticoagulant properties of proteins C and S, disrupt the cell surface annexin A5 sheath [37], inhibit VWF-dependent platelet aggregation [38], and complement activation [39]. Thus, the results of our and other studies confirm the purposefulness of searching for APLA in explaining the etiopathogenic factors of CRAO.

In our study, the prevalence of elevated FVIII activity was similar in both groups. However, its absolute measures were higher in CRAO patients. Surprisingly, results on elevated FVIII in CRAO patients are unconstant. Some of them indicated higher activity [3, 19], while the others showed no differences [18]. Intriguingly, FVIII is perceived as a risk factor for venous and arterial thrombosis, as its raised values are associated with enhanced coagulation cascade and unfavorable cardiovascular complications [40]. Nevertheless, our study group had significantly higher FVIII activity. Thus, elevated FVIII levels may somehow play a role in the pathogenesis of CRAO.

The prevalence of hyperhomocysteinemia in our CRAO group was significantly higher than in controls, which stays in line with other reports [3, 19, 23, 24] and meta-analyses on RAO patients [22, 35]. Higher homocysteine is claimed to be a cardiovascular risk factor, which may exaggerate the prothrombotic state, damage the vascular endothelium and enhance oxidative stress [41]. Although its levels depend on dietary habits, comorbidities, or medications, hyperhomocysteinemia is a partially treatable risk factor as the supplementation of folic acid and B-vitamin can normalize its levels [42]. Nonetheless, it is worth highlighting that treatment with vitamins, even if efficiently decreasing homocysteine concentrations, does not lower the cardiovascular risk [43].

According to genes variants, FVL mutation was more frequent in the CRAO group than in controls. Surprisingly, this result stays in contrast with other research papers on this topic [3, 19, 20, 25, 44]. Nevertheless, the study by Nagy et al. [18] showed that the occurrence of FVL almost four times significantly increased the risk of RAO development compared with controls. The overall occurrence of FVL in a meta-analysis by Romiti et al. [35] based on almost three hundred RAO patients is estimated with a fixed effect model to be around 6%, which is lower than observed in our study group. On the contrary, our data on the prothrombin G20210A genetic variant mirror those published previously on RAO [3, 18, 19, 25, 44]. The prevalence of this genetic variant in our group is much higher than 3% in a meta-analysis of RAO patients [35]; however, similar to controls, which may reflect our Polish population.

The next issue that needs comment is the role of factor XIII (FXIII) in the CRAO, which has not been deeply studied in RAO patients so far. Indeed, FXIII is a plasma transglutaminase that plays a role in fibrin stabilization. The Val34Leu polymorphism of FXIII results from a substitution of amino acid leucine (Leu) to valine (Val) at position 34 in the FXIII molecule. Interestingly, this polymorphism was previously reported as associated with a lower risk of stroke, myocardial infarction, and deep vein thrombosis [45]. Moreover, a study by Weger et al. [46] in a cohort of 108 RAO patients demonstrated that the homozygous Leu genotype was less frequent in the patients. Therefore, the authors concluded that the Leu/Leu genotype might be associated with a protective role against RAO. However, our results contrast with that report in the CRAO group.

Currently, thrombophilic screening in CRAO patients is not recommended in each patient due to inconsistent data on its prevalence [35, 47]. However, our data indicate that such testing needs to be advised, particularly regarding APLA. Furthermore, in APS, we must implement proper anticoagulant management, using treatment with vitamin K antagonists and acetylsalicylic acid, to prevent another thromboembolic complication [48]. Interestingly, therapy with direct oral anticoagulant inhibitors (DOAC) is not currently recommended in APS patients, despite its benefits in other indications [49]. Therefore, we believe screening for thrombophilic risk factors should be highly considered and recommended in all CRAO patients. Hence, implementing proper approaches, including preventive (managing with traditional risk factors) and therapeutic (anticoagulant/antiplatelet treatment) ones, may limit further complications. Nevertheless, a multidisciplinary approach to diagnosing and treating ophthalmologic thrombotic complications is crucial in understanding CRAO etiopathogenesis.

### Study limitation

Firstly, all coagulatory variables were measured once, and we cannot exclude their changes over time. Patients and control groups differed according to comorbidities; however, statistical analyses were performed with adjustments for those confounders, and they likely have no impact on our results. Furthermore, subgroup analysis needs to be interpreted with caution as, in some cases, it may be incidental and not represent cause-and-effect relationships.

## Conclusions

Our results in a comprehensive way showed that CRAO patients, compared with controls without thromboembolic events in medical history, are characterized by having laboratory and genetic thrombophilic risks factors, including higher prevalence of protein C deficiency, lower free protein S levels, presence of antiphospholipid antibodies, factor V Leiden mutation, and hyperhomocysteinemia. Thus, detecting the above-mentioned unfavorable factors might be beneficial in identifying subjects with an increased risk of future thromboembolic events with individual approaches to avoid severe cardiovascular complications.

## Data Availability

The data presented in this study are available upon reasonable request from the corresponding author.
